# Waste analysis and energy use estimation during MR-HIFU treatment: first steps towards calculating total environmental impact

**DOI:** 10.1186/s13244-024-01655-2

**Published:** 2024-03-22

**Authors:** Kimberley J. Anneveldt, Ingrid M. Nijholt, Joke M. Schutte, Wouter J. K. Hehenkamp, Sebastiaan Veersema, Judith A. F. Huirne, Martijn F. Boomsma

**Affiliations:** 1 Department of Radiology, Isala Hospital, Dokter Van Heesweg 2, Zwolle, 8025 AB The Netherlands; 2 Department of Reproductive Medicine and Gynecology, University Medical Center Utrecht, Heidelberglaan 100, Utrecht, 3584 CX The Netherlands; 3 Image Sciences Institute, Division of Imaging & Oncology, University Medical Centre Utrecht, Heidelberglaan 100, Utrecht, 3584 CX The Netherlands; 4 Department of Gynecology, Isala Hospital, Dokter Van Heesweg 2, 8025 AB Zwolle, The Netherlands; 5 Department of Obstetrics and Gynecology, Amsterdam University Medical Centre, Location AMC, Meibergdreef 9, Amsterdam, 1105 AZ The Netherlands; 6 Department of Obstetrics and Gynecology and Amsterdam Research Institute Reproduction and Development, Amsterdam University Medical Centre, Location AMC, Meibergdreef 9, Amsterdam, 1105 AZ The Netherlands

**Keywords:** Leiomyoma, MRI, Program evaluation, Ultrasonography

## Abstract

**Objectives:**

To assess the environmental impact of the non-invasive Magnetic Resonance image-guided High-Intensity Focused Ultrasound (MR-HIFU) treatment of uterine fibroids, we aimed to perform a full Life Cycle Assessment (LCA). However, as a full LCA was not feasible at this time, we evaluated the CO_2_ (carbon dioxide) emission from the MRI scanner, MR-HIFU device, and the medication used, and analyzed solid waste produced during treatment.

**Methods:**

Our functional unit was one uterine fibroid MR-HIFU treatment. The moment the patient entered the day care-unit until she left, defined our boundaries of investigation. We retrospectively collected data from 25 treatments to assess the CO_2_ emission based on the energy used by the MRI scanner and MR-HIFU device and the amount and type of medication administered. Solid waste was prospectively collected from five treatments.

**Results:**

During an MR-HIFU treatment, the MRI scanner and MR-HIFU device produced 33.2 ± 8.7 kg of CO_2_ emission and medication administered 0.13 ± 0.04 kg. A uterine fibroid MR-HIFU treatment produced 1.2 kg (range 1.1–1.4) of solid waste.

**Conclusions:**

Environmental impact should ideally be analyzed for all (new) medical treatments. By assessing part of the CO_2_ emission and solid waste produced, we have taken the first steps towards analyzing the total environmental impact of the MR-HIFU treatment of uterine fibroids. These data can contribute to future studies comparing the results of MR-HIFU LCAs with LCAs of other uterine fibroid therapies.

**Critical relevance statement:**

In addition to (cost-) effectiveness, the environmental impact of new treatments should be assessed. We took the first steps towards analyzing the total environmental impact of uterine fibroid MR-HIFU.

**Key points:**

• Life Cycle Assessments (LCAs) should be performed for all (new) medical treatments.

• We took the first steps towards analyzing the environmental impact of uterine fibroid MR-HIFU.

• Energy used by the MRI scanner and MR-HIFU device corresponded to 33.2 ± 8.7 kg of CO_2_ emission.

**Graphical Abstract:**

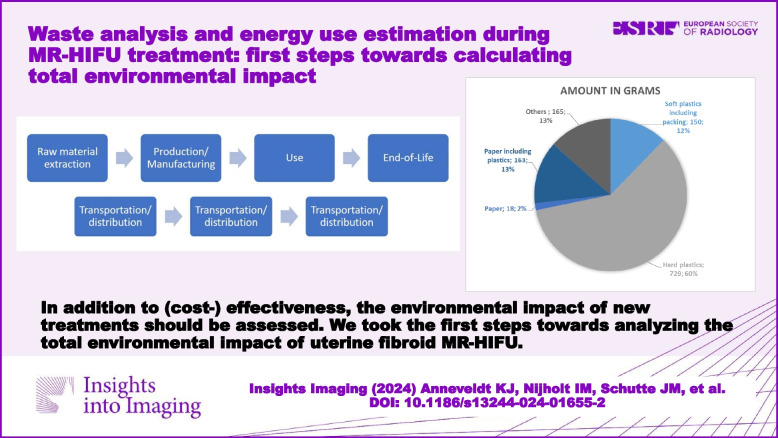

## Introduction

Healthcare is responsible for nearly 8% of carbon dioxide (CO_2_) emissions in the United States each year [1, 2]. It follows that, paradoxically, healthcare contributes to poorer health by producing CO_2_ which acts as one of the greenhouse gases causing global warming. Reducing healthcare’s impact on the environment is therefore among the greatest challenges facing healthcare in the twenty-first century [1, 3, 4].

Fortunately, measuring and reducing the environmental impact of medical practices is gaining attention [5]. The number of Life Cycle Assessments (LCAs) providing information on the environmental impact of healthcare has grown rapidly [6].

**Fig. 1:**
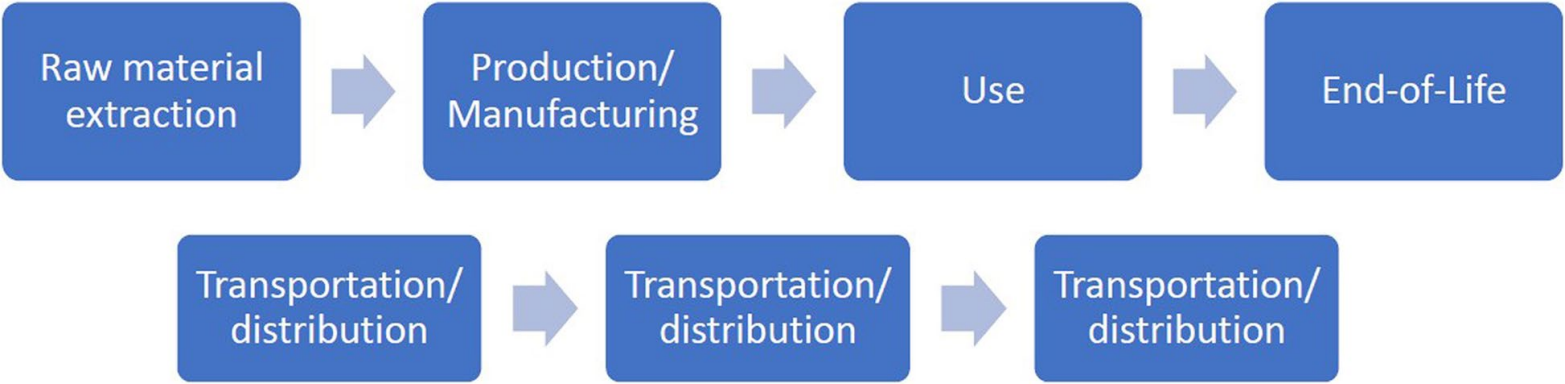
Life cycle phases of product, process, or system

LCA is a methodology to quantify a multifactorial range of environmental impact categories (e.g., global warming), associated with the full life cycle of products, processes, and systems analyzed together [5, 7]. The main goal of an LCA is to be as complete as possible by covering each phase of the life cycle of a product, process, or system. This approach is often referred to as a cradle-to-grave analysis (Fig. 1). The life cycle phases are generally categorized as (raw) material extraction, manufacturing, use, and disposal and additionally include transportation between each phase.

**Fig. 2:**
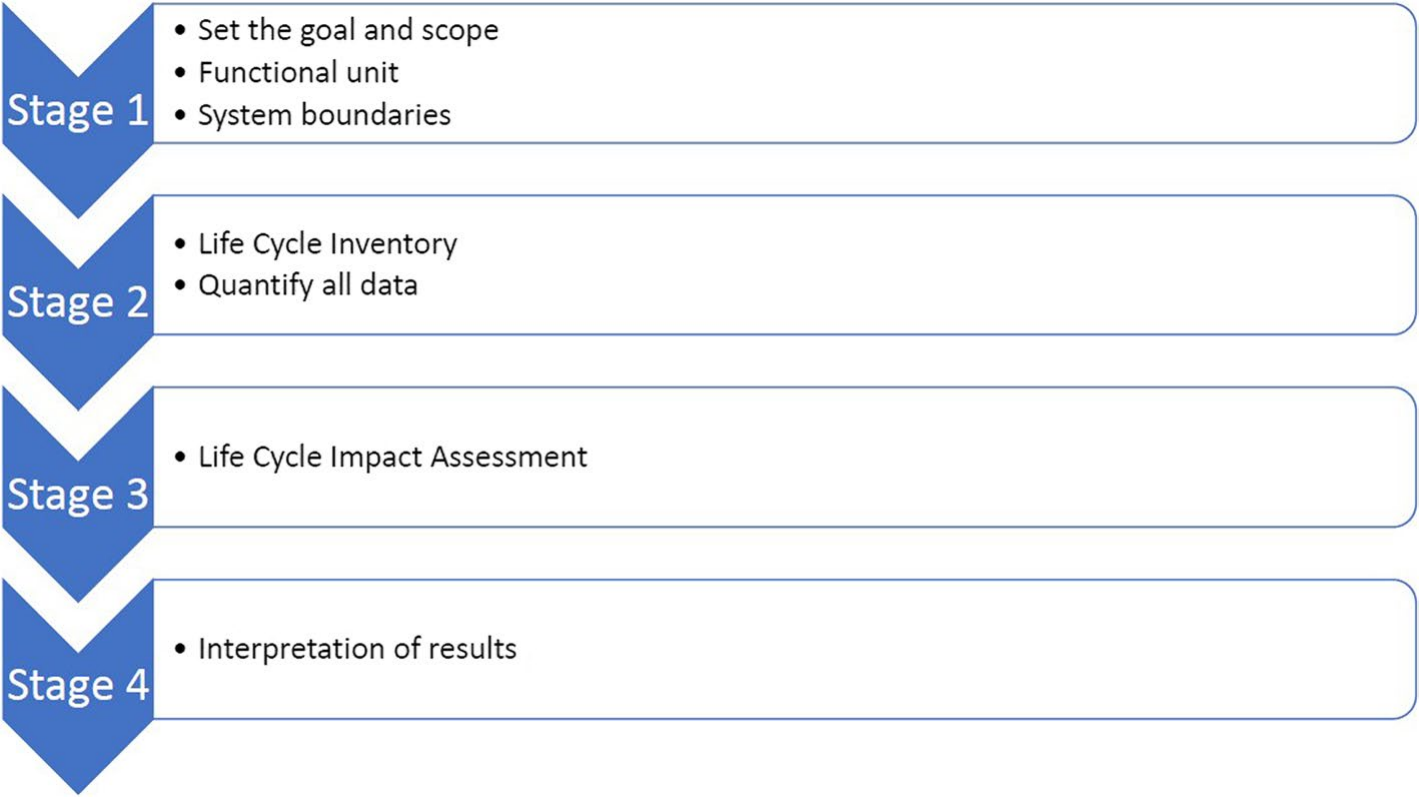
The four stages of a Life Cycle Assessment

The principle of an LCA includes four stages (Fig. 2). In the first stage, goal(s) and scope are determined, defining the functional unit (the product, process, or system to be analyzed) and system boundaries. As it is often not feasible to include every element of each life cycle phase, a detailed description and the justification of the system boundaries are of great importance. In the second stage, a Life Cycle Inventory (LCI) is performed. Data on all materials and energy inputs and outputs are collected and quantified for the functional unit [8]. Primary data is preferred for this collection, but secondary data is often used [5]. The second stage answers the question of how many materials and how much energy are required. In the third stage, the Life Cycle Impact Assessment (LCIA), the potential environmental impact, per category and in total, of these materials and energies is determined. This provides an answer to the question of how harmful the inventory items are. The fourth and final stage is to interpret the results [9].

In 2016, (interventional) radiologists started performing the relatively new non-invasive Magnetic Resonance image-guided High-Intensity Focused Ultrasound (MR-HIFU) treatment for uterine fibroids at our hospital. MR-HIFU uses a focused ultrasound beam to ablate tissue under MRI guidance [10]. Advantages of this treatment over other uterine fibroid treatments include its non-invasive nature, short recovery time, and low incidence of adverse events [10, 11]. Currently, this treatment is not reimbursed in the Netherlands. To obtain reimbursement, an RCT on (cost-) effectiveness is currently being conducted [12]. However, keeping the impact of healthcare on the environment in mind, the environmental impact of this relatively new treatment should also be analyzed.

Studies have been done on environmental impacts within the field of radiology; however, full LCAs of (interventional radiology) treatments have not yet been performed [13]. The main focus has been on the energy consumption of different diagnostic modalities and how it can be reduced [14–16], on identifying the types of waste generated and how it can be reduced, and on understanding potential barriers to implementing green initiatives [17].

The current standard of care for uterine fibroids includes hormonal treatment, interventional radiology procedures, and surgical management [18]. Within gynecology, the environmental impact of its practices is a topic that is gaining increasing attention and has resulted in several publications [19]. For example, LCAs have compared the carbon footprint of different materials of vaginal specula or delivery sets [20–22].

Hitherto, no LCA has been performed for any MR-HIFU indication, nor uterine fibroid treatment [19]. Due to a lack of resources on our part, and the general lack of an available LCA database including all necessary healthcare products, it was not feasible to perform a full LCA. Therefore, we aimed to perform the first steps of analyzing the environmental impact of MR-HIFU for uterine fibroids by including components that we could analyze: estimating CO_2_ emission of the energy consumed by the MR scanner and the MR-HIFU device, the medication administered, and a waste analysis during an MR-HIFU treatment. Furthermore, we describe which steps are necessary to be able to perform a full LCA, and why and which actions are needed within the interventional radiology community to achieve this.

## Methods

### Stage 1: Goal, scope, and system boundaries

**Fig. 3:**
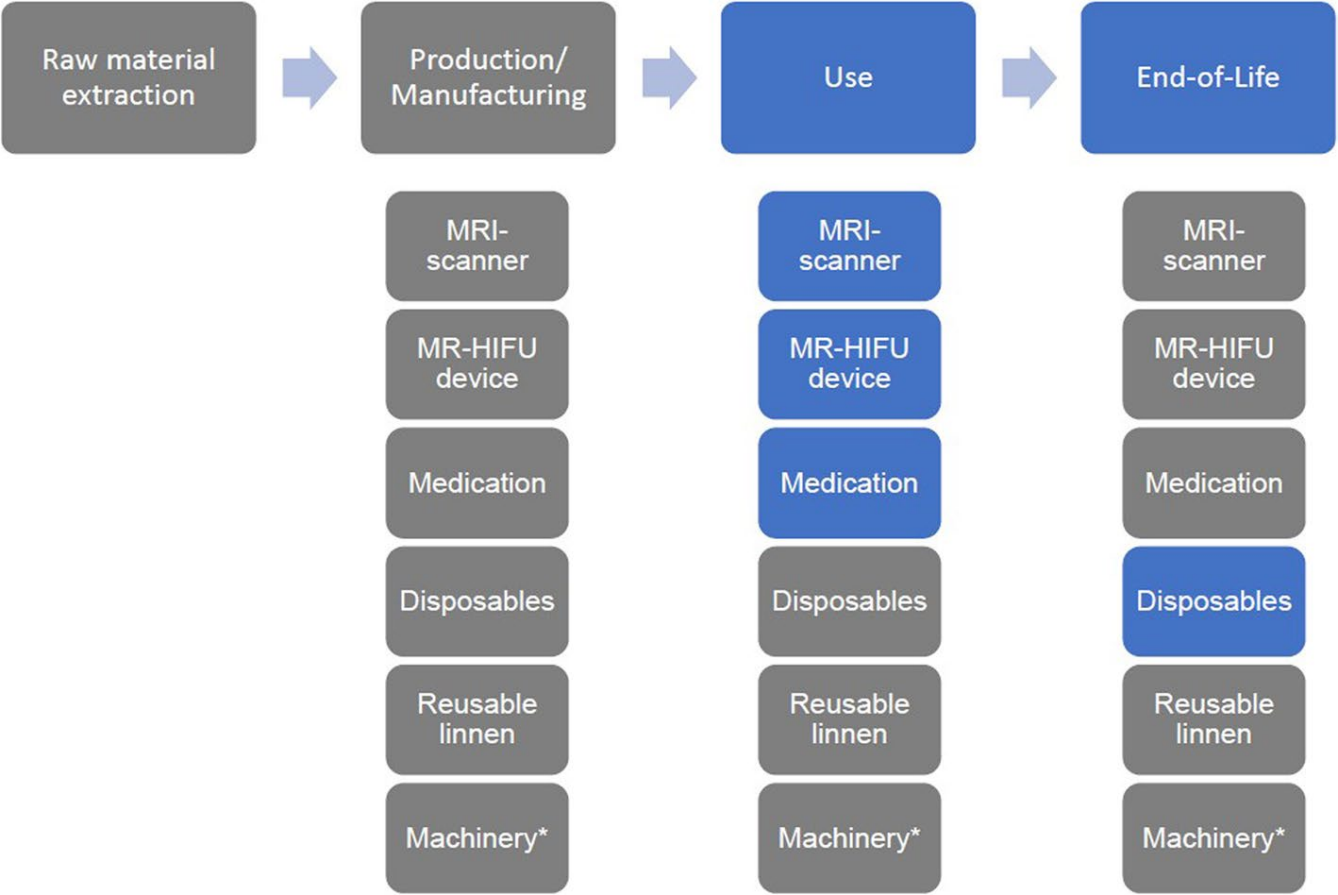
Life cycle of MR-HIFU treatment. *Computers and devices used by the radiology and anesthesiology department, e.g., blood pressure, monitor. Gray: excluded. Blue: included in this study

Our Local Medical Ethical Committee waived this research (study number 20230334). The functional unit was one technically successful uterine fibroid MR-HIFU treatment performed on the latest version of the CE-marked Sonalleve MR-HIFU platform (Profound Medical Corp., Canada) integrated into a 1.5-T MR scanner (Achieva; Philips Healthcare, the Netherlands) [10]. We analyzed three components of this treatment: (1) energy used by the MRI scanner and MR-HIFU device during an MR-HIFU treatment, (2) the medication used during treatment and admission, and (3) solid waste produced during treatment and admission (Fig. 3). Data were retrospectively collected from 25 uterine fibroid MR-HIFU treatments in the radiology department of our hospital between September 2020 and January 2022. All treatments were performed by radiologists with similar experience of more than 4 years. Due to the retrospective aspect, the operators were not aware of the study.

### Stage 2: Life Cycle Inventory

#### Energy consumption of MRI scanner

To assess the energy used by the MRI scanner, the average total treatment duration of 25 treatments (time between first T2-survey MRI sequence and last T1w-CE MRI sequence) and the average duration of the active and idle states were determined (Table 1). The duration of ablation was calculated by adding up the duration of all individual sonications during a treatment. The duration of the idle state of the MRI scanner was calculated by subtracting the average duration of all MRI sequences applied during a treatment (T2-survey, skin bubble, DWI, T2-planning, and T1w-CE) and the duration of ablation from the total treatment duration. Subsequently, the energy consumption of the MRI scanner was calculated per mode by multiplying the duration of a given state by the peak kW, using data from a previous report by Walthery [23]. He assumed that a general 1.5 T Philips MRI scanner uses 17 kW when active and 12 kW in an idle state. An additional 33% kW should be added to the total, to cover energy usage by the cooling system. The energy consumed during ablation was expected to be comparable to the active state.

**Table 1:** Overview of mean power, mean duration, mean and standard deviation energy use, and mean and standard deviation CO_2_ emission of MRI scanner and MR-HIFU device status during a uterine fibroid MR-HIFU treatment

	**Power (kW)**	**Mean duration (minutes)**	**Energy use (kWh)**	**CO2 emission (kg)**
**1.5-T MRI scanner**				
Idle	12	149	29.7 ± 8.2	
Active	17	30	8.5 ± 2.4	
Ablation (active)	17	24	6.9 ± 3.4	
Additional energy use 33%	n.a.	n.a.	14.9 ± 3.9	
**Total**			**60.0 ± 15.6**	**31.4 ± 8.2**
**MR-HIFU device**				
Active	11	24	1.0 ± 0.5	
Idle	1	178	2.5 ± 0.6	
**Total**			**3.5 ± 1.1**	**1.8 ± 0.6**
**MRI scanner and device**				
**Total**			**63.5 ± 16.7**	**33.2 ± 8.7**

*CO2* carbon dioxide, *MR-HIFU* Magnetic Resonance image-guided High-Intensity Focused Ultrasound, *kW* kilowatt, *kWh* kilowatt-hour, *n.a.* not applicable

#### Energy consumption of MR-HIFU device

The manufacturer of the Sonalleve device provided us with the energy consumed during the active and idle states of the MR-HIFU device. There is currently no public source available to confirm this data. The energy used in the idle state is 844 W. The additional energy used during ablation depends on the selected power by the treatment provider and should be multiplied by 11 to calculate the total energy used during ablation. To calculate the average total energy consumed by the MR-HIFU device during treatment, the average duration of idle state and ablation was multiplied by the energy consumed during the active and idle states of the MR-HIFU device, respectively.

#### Medication

All oral and intravenous medications administered to the patient during treatment and admission were collected to calculate an average use per treatment, together with the amount of oxygen applied by the nasal cannula. These data were retrieved from the electronic patient file, which showed which medication was administered at what moment with what duration.

#### Waste audit

All disposables during five consecutive treatments in March and April 2022 were collected, sorted, and weighed by the researcher (K.A.) on the treatment day after the discharge of the patient. The treatment team on that day participated by depositing their waste in designated waste bins. Packaging materials of sharp materials were included; sharp materials themselves were excluded. Packaging of medication (e.g., glass flacons) was included after emptying. All waste was weighted in total and per five waste types, i.e., soft plastics including packaging, hard plastics, paper, paper including plastics, and others.

### Stage 3: Life Cycle Impact Assessment

For this analysis, only the environmental impact category CO_2_ emission (kg) was selected, for which we used the conversion factor “grey energy” (0.523 kg CO_2_/kWh) to convert kWh energy [24]. Greenhouse gas emissions for six anesthetic drugs and/or painkillers were retrieved from previous studies [6, 25]. For the remaining medications, an average of 340 g CO_2_ emission/g drugs was used [25, 26].

### Statistical analysis

Statistical analyses were performed using IBM SPSS version 26. Continuous variables were presented as mean (± SD) in the case of a normal distribution. Distribution was assessed by normal probability plots and eyeball testing. A 95% confidence interval was also presented.

## Results

### Energy consumption

The average energy required by the MR-HIFU device during ablation was 150.0 ± 27.8 W 95% CI (138.4, 161.4). The average total energy used was 60.0 ± 15.6 kWh 95% CI (53.5, 66.5) from the MRI scanner and 3.5 ± 1.1 kWh 95% CI (3.1, 4.0) from the MR-HIFU device per treatment (Table 1). This resulted in a CO_2_ emission of 31.4 ± 8.2 kg 95% CI (28.0, 34.8) by the MRI scanner and 1.8 ± 0.6 kg 95% CI (1.6, 2.1) by the MR-HIFU device. In total, 33.2 ± 8.7 kg 95% CI (29.6, 36.8) CO_2_ emission was produced per MR-HIFU treatment.

### Medication

Eleven types of medication were administered to at least five patients. Six additional types of medication were administered only once and were excluded from analyses (Table 2). All included medication administered during a uterine fibroid MR-HIFU treatment totaled 0.13 ± 0.04 kg 95% CI (0.11, 0.14) of CO_2_ emission.

**Table 2:** Medication use during the MR-HIFU treatment of uterine fibroids

Name, dosage, route	Average (number of patients (*N*) /percentage)	Range [min–max]	Conversion factor (gCO2/g)	CO2-emission (kg)
Propofol 2%, perfusor infusion	392.6 mg (25, 100%)	60.36–1000 mg	21	0.0083
Fentanyl 25–100 µg/mL	162.0 µg (23, 92%)	25–550 µg	96	> 0.000
Lidocaine 2%	17.2 mg (16, 64%)	10–40 mg	29	0.0005
Carbetocine 100 µg/mL	88.0 µg (22, 88%)	0–100 µg	340	> 0.000
Gadoteeracid 7.5 mmol/15 mL	7.5 mmol (25, 100%)	7.5 mmol	340	0.0026
Natriumchloride 0.9% perfusor infusion	266.2 mg (25, 100%)	116.25–450.00 mg	200	0.053
Paracetamol, 500 mg tablet	1420.0 mg (24, 96%)	1000–3000 mg	7.8	0.011
Diclofenac, 50 mg tablet	90.0 mg (20, 80%)	50–200 mg	340	0.031
Oxycodon short-acting, 10 mg meltingtablet	10.8 mg (24, 96%)	10–30 mg	340	0.004
Microlax, 5 mL sachet^a^	4.4 mL (22, 88%)	0–5 mL	x	x
Granisetron 1 mg/mL	0.2 mg (5, 20%)	0–1 mg	340	> 0.000
Oxygen 2 L	221.1 min (24, 96%)	163–345 min	2.1	0.016
Buscopan 10 mg/0.5 mL^b^	0.4 mg (1, 4%)	0–10 mg		x
Pantoprazol 40 mg tablet^b^	1.5 mg (1, 4%)	0–40 mg		x
Atropine, 0.5 mg/mL^b^	> 0.0 mg (1, 4%)	0–0.5 mg		x
Dexamethason 4 mg/mL^b^	0.2 mg (1, 4%)	0–4 mg		x
Alfentanil 0.25 mg/0.5 mL^b^	0.1 mg (1, 4%)	0–0.25 mg		x
**Total per patient**				**0.125 ± 0.04**

*MR-HIFU* Magnetic Resonance image-guided High-Intensity Focused Ultrasound, *gCO2/g* gram carbon dioxide per gram

^a^Combination medication, excluded from the analysis

^b^Medication applied to only one patient, excluded from the analysis

### Waste audit

**Fig. 4:**
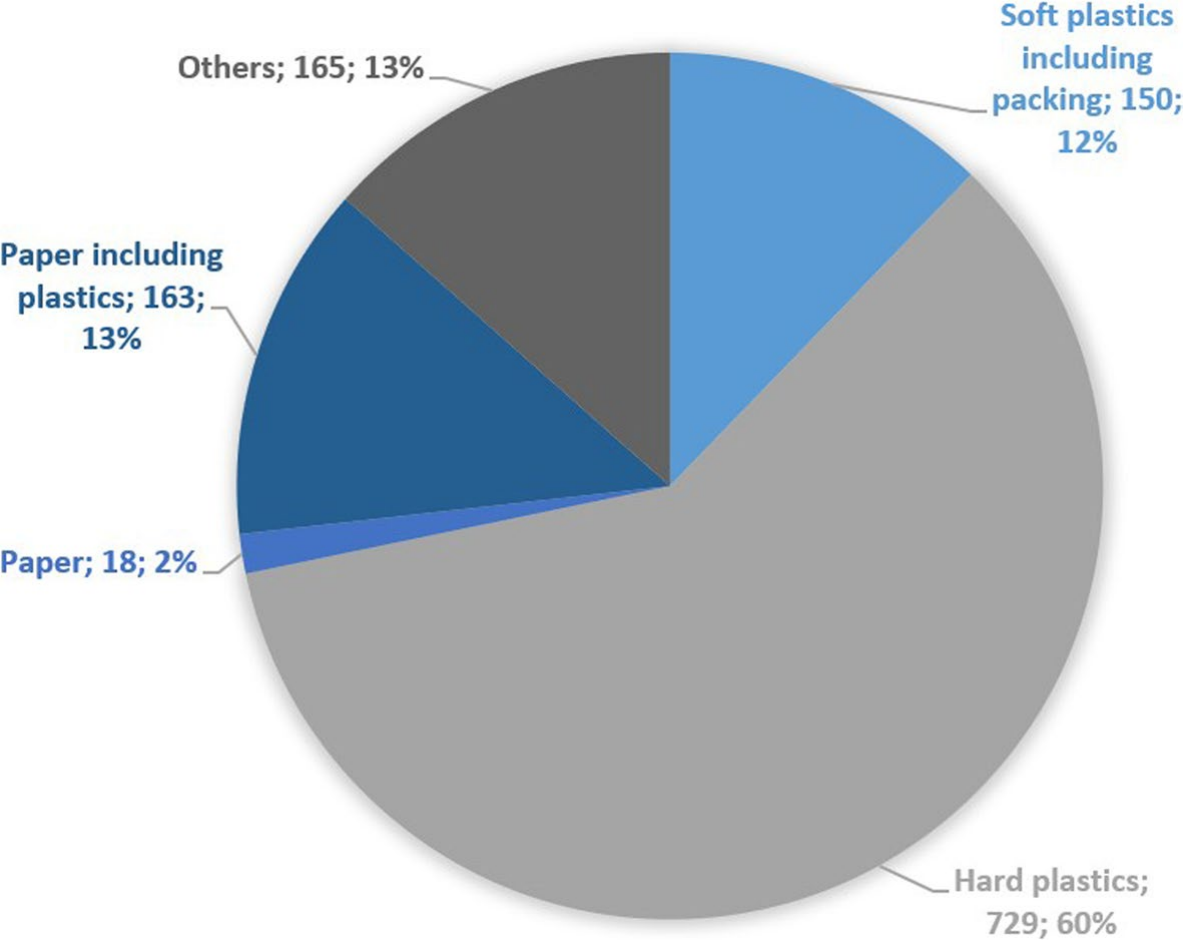
Different types of waste after waste audit. Broken down into the type of waste, amount (in grams), and percentage of total

The mean weight of the solid waste was 1.2 kg (range = 1.1–1.4) (Fig. 4).

## Discussion

Despite the major impact of healthcare on the environment, the sustainability of (new) treatments is currently understudied. We took the first steps towards analyzing the environmental impact of an MR-guided interventional radiology treatment, i.e., the MR-HIFU treatment of uterine fibroids, by evaluating the CO_2_ emission of energy use by the MRI scanner and the MR-HIFU device, the CO_2_ emission associated with medication use, and by evaluating the amount of solid waste produced during a single treatment. We are aware that our results do not represent a full LCA of uterine fibroid MR-HIFU and that they underestimate the total use. In an ideal situation, different environmental impact categories would be part of an LCA and all elements of the MR-HIFU treatment life cycle would be analyzed. Nevertheless, we believe publishing this data is important as it may contribute to future comparative LCAs. More importantly, with this study, we aimed to underline the need to support and perform LCAs for treatments, and to provide further insight into the challenges of performing an LCA in a healthcare setting, and specifically in interventional radiology [27, 28].

### Energy consumption

The operating mechanism of an MR-HIFU treatment is induced focused energy. Therefore, the energy consumed by the MR-HIFU device is an important contributor to total CO_2_ emission during treatment. In our analyses, we used the conversion factor “grey energy,” a representative Dutch combination of coal, gas, and nuclear energy, without considering the energy used to build the production facilities [24]. The emission of kWh energy generated by water, wind, or solar energy is 0.004, 0.014, and 0.061 kg CO_2_/kWh, respectively [24]. In our hospital, all energy is CO_2_ neutrally generated. Therefore, the exact amount of energy needed to perform the MR-HIFU treatment may seem less relevant. However, even “green” energy must be generated and may have an impact on other environmental impact categories.

### Medication

The CO_2_ emission of pharmaceuticals is understudied, and industry LCA publications cannot be verified, as they require access to confidential manufacturing processes [1, 29]. Medication accounts for around 25% of the UK’s total healthcare CO_2_ emissions [30].

To estimate the CO_2_ emission of the medication administered during an MR-HIFU treatment, we used CO_2_ emission data from previous studies. These studies often did not include the emission of packaging [6, 25, 26]. McAlister et al. calculated that 90% of morphine CO_2_ emissions were caused by sterilization and packaging. Therefore, sterilization and packaging should not be neglected [29]. An important advantage of MR-HIFU treatment over uterine fibroid surgery is that there is no need for anesthetic gases, which are a major contributor to the CO_2_ emissions from medication in general [6, 30].

### Waste audit

Hospitals in the USA generate 3.4 billion pounds of solid waste annually [7]. The procurement supply chain causes most of the CO_2_ emission. Therefore, decisions made at the product manufacturing stage could reduce environmental impact. Clements et al. analyzed the packaging waste from single-use products used for interventional radiology procedures [31]. Of the 72 products analyzed, 55% of their total weight consisted of waste and 76% could potentially be safely replaced by reusable products. We did not include the amount of recyclability in the scope of our research because no recycling policy was available in our hospital, or in the healthcare system on interventions, but this aspect should be included in future analyses.

### LCAs on uterine fibroid treatments

Ideally, a comparative study would have been conducted, comparing LCAs of different uterine fibroid treatments. However, at this time, such a study is not feasible due to several hurdles, including the lack of an LCA database with relevant health inventories, the lack of standardization of how a healthcare treatment LCA should be performed, and what boundaries are considered acceptable. As a result, the only studies available at this stage are those such as ours that include only part of an environmental analysis. Thiel et al. performed a waste analysis after different types of hysterectomy with an average mass of 13.7 kg—much higher than the 1.2 kg we collected after MR-HIFU [7]. Chua et al. performed an LCA of all energy, materials, and waste used in a radiological intervention room [32], reporting on 98 interventions including embolizations, but the indications for these procedures were unclear and they may not have been performed as treatment of uterine fibroids. For future comparisons, it is necessary to standardize which elements of the treatments should be included and to provide as disaggregated data as possible.

### Limitations

The main limitation of our study is that we did not perform a full cradle-to-grave LCA of uterine fibroid MR-HIFU and therefore it should be considered as preliminary. Ideally, all life cycle phases of a product, process, or system are included since interventions in one phase may have consequences in another [5]. At the same time, all LCA studies are incomplete to some extent because boundaries must be set to limit the amount of data and analysis required. We only included the three components of the MR-HIFU treatment (Fig. 3) for which we had the resources to examine: those that were in our own center of influence and were expected to differ most from other uterine fibroid treatments. Current LCA databases lack data on healthcare processes, products, and systems data and, together with LCIA software, are not publicly available. The reason for this seems to be a lack of awareness and transparency by vendors, hospital mechanics, and energy suppliers. This hurdle could be overcome if sustainability became as important as clinical effectiveness and cost-effectiveness in healthcare.

Unfortunately, it was not possible to measure the energy consumed by both the MR scanner and the MR-HIFU device during the MR-HIFU treatment. Therefore, we used the second-best option, which is often done in LCAs and concerns literature sources. It is important to note that these literature sources are limited and often contain unvalidated data. We included energy consumption data from a comparable MRI scanner that we used during our treatments from a master’s thesis [23]. In other publications, different MRI scanners were used, but the amount of energy per mode was comparable and therefore considered legitimate [13, 14].

Moreover, the amount of energy calculated in this study is an underestimation of the total energy used. For example, the use of heating, ventilation, and air conditioning used in the MRI room can also be relevant contributors [32]. The production of the MRI scanner was excluded from this analysis. Since the MRI scanner has an average lifespan of 15 years, it is questionable whether the emission from the construction of the MRI scanner contributes significantly to the CO_2_ emission of a single treatment [13].

For medication use, the main limitation is the fact that, for most drugs, there is no LCA data available or is it not possible to retrieve such data due to drug patents. Furthermore, we did not analyze the amount of unused medication. Unused and disposed medication is an important contributor to the negative environmental impact of healthcare in general and, if not correctly disposed of, can contribute to water contamination [4].

Our amount of waste measured is most likely an underestimation, as we did not include sharp materials, although the additional weight is expected to be small. In addition, since the waste analyses were prospective, bias due to the treatment team’s knowledge of the amount of waste produced could not be ruled out. However, the team involved in the MR-HIFU treatments was not specifically engaged with sustainability.

### Future perspectives

Timely action is urgently needed to reduce the environmental impact of healthcare. In light of this study and the challenges that remain, we would make some suggestions for change so that the interventional radiology community can take responsibility and make a positive contribution.

First, we should determine the appropriate indication for treatment and treat only when necessary and beneficial. We should minimize the use of materials, substitute them with more environmentally friendly products, move away from certain heat-trapping anesthetic gases, maximize instrument reuse or encourage (research into) re-usable instruments, and reduce off-hour energy consumption [7, 21, 22, 31, 33]. Secondly, the hospital purchasing departments need to focus on the environmental impact of the products they purchase, and hospitals should collaborate with suppliers who are willing to provide information on the environmental impact of their products; authorities and hospitals should feel the societal responsibility to be part of this change.

Thirdly, we should feel the need to perform LCAs and should be willing to contribute to them. Standardizing the way in which LCAs of treatments in healthcare are carried out, including clarity on how boundaries are set, should be developed by the community and high-quality healthcare LCA databases and LCIA software should be made available on an open-access basis. To create this healthcare database, the community as a whole should be willing to work open access and it should become a mandatory element of future funding and grants. Finally and importantly, research grants focused on sustainability could contribute to the transformation process. They could provide the much-needed financial contribution to support the necessary actions outlined above. In the Netherlands, a specific healthcare LCI database is currently being developed with government funding.

## Conclusion

We took the first steps within MR-guided interventional radiology towards analyzing the environmental impact of a complete treatment—the MR-HIFU treatment of uterine fibroids. Energy consumption of the MRI scanner and MR-HIFU device resulted in 33.2 kg of CO_2_ emission and medication administered contributed 0.13 kg of CO_2_ emission. Moreover, 1.2 kg solid waste was collected. Full LCAs need to be performed to make a definitive comparison of the environmental impact of different uterine fibroid treatments. By adopting this approach, the interventional radiology community could take responsibility for reducing its impact on climate change.

## Data Availability

Anonymized collected data is available on reasonable request. Data cannot be found on any publicly available database.

## Abbreviations

CO_2_: Carbon dioxide
LCA: Life Cycle Assessment
LCI: Life Cycle Inventory
LCIA: Life Cycle Impact Assessment
MR-HIFU: Magnetic Resonance image-guided High-Intensity Focused Ultrasound
